# Lack of association between the *CARD10* rs6000782 polymorphism and type 1 autoimmune hepatitis in a Japanese population

**DOI:** 10.1186/s13104-015-1733-4

**Published:** 2015-12-12

**Authors:** Kiyoshi Migita, Yuka Jiuchi, Hiroshi Furukawa, Minoru Nakamura, Atsumasa Komori, Michio Yasunami, Hideko Kozuru, Seigo Abiru, Kazumi Yamasaki, Shinya Nagaoka, Satoru Hashimoto, Shigemune Bekki, Kaname Yoshizawa, Masaaki Shimada, Hiroshi Kouno, Hiroshi Kamitsukasa, Tatsuji Komatsu, Taizo Hijioka, Makoto Nakamuta, Atsushi Naganuma, Haruhiro Yamashita, Hideo Nishimura, Hajime Ohta, Yoko Nakamura, Keisuke Ario, Yukio Oohara, Kazuhiro Sugi, Minoru Tomizawa, Takeaki Sato, Hironao Takahashi, Toyokichi Muro, Fujio Makita, Eiji Mita, Hironori Sakai, Hiroshi Yatsuhashi

**Affiliations:** NHO-AIH study group, Nagasaki Medical Center, Kubara 2-1001-1, Omura, Nagasaki 856-8562 Japan; Department of Rheumatology, NHO Sagamihara Hospital, Minamikusakuradai 18-1, Sagamihara, Kanagawa 252-0392 Japan; Department of Hepatology, Nagasaki University Graduate School of Biomedical Sciences, Sakamoto 1-12-4, Ngasaki, Nagasaki 852-8523 Japan; Department of Clinical Medicine, Institute of Tropical Medicine, Nagasaki University, Sakamoto 1-12-4, Ngasaki, Nagasaki 852-8523 Japan; Clinical Research Center, NHO Nagasaki Medical Center, Kubara 2-1001-1, Omura, 856-8652 Japan

**Keywords:** Autoimmune hepatitis, Genetic factor, Genome-wide association study, CARD10

## Abstract

**Background:**

Previous genome-wide association studies have evaluated the impact of common genetic variants and identified several non-HLA risk loci associated with autoimmune liver diseases. More recent genome-wide association studies and replication analyses reported an association between variants of the *CARD10* polymorphism rs6000782 and risk of type 1 autoimmune hepatitis (AIH). In this case–control study, we genotyped 326 Japanese AIH patients and 214 control subjects.

**Results:**

Genomic DNA from 540 individuals of Japanese origin, including 326 patients with type-1 AIH and 214 healthy controls, was analyzed for two single nucleotide polymorphisms (SNPs) in the *CARD10* gene. We selected *CARD10* rs6000782 SNPs and genotyped these using PCR–RFLP method and direct sequencing. The Chi square test revealed that the rs6000782 variant alle (c) was not associated with the susceptibility for AIH in a Japanese population [*p* = 0.376, odds ratio (OR) 1.271, 95 % confidence interval (CI) 0.747–2.161] in an allele model. Our data also showed that *CARD10* rs6000782 variants were not associated with AIH or with the clinical parameters of AIH.

**Conclusions:**

In this study we examined an association between rs6000782 SNPs in the *CARD10* gene and type-1 AIH. Results showed no significant association of rs62000782 with type-1 AIH in a Japanese population. This study demonstrated no association between *CARD10* rs6000782 variants and AIH in a Japanese population.

## Background

Autoimmune hepatitis (AIH) is characterized by the presence of serum antibodies, both anti-nuclear (ANA) and anti-smooth muscle antibodies (ASMA), as well as elevated immunoglobulin G levels, and interface hepatitis [1]. The genetic factors underlying the occurrence of AIH are unknown, with the exception of certain human leukocyte antigen (HLA) alleles [2]. de Boer et al. previously conducted a genome-wide association study that identified the most prominent association with AIH at rs2187668, which maps to the intronic region of *HLA*-*DQA1* [3]. They also showed that AIH was associated with variants of genes encoding Scr homology 2 adaptor protein 3 (*SH2B3*; rs3184504) and caspase recruitment domain-containing protein 10 (*CARD10*; rs6000782) [3]. In view of the importance of understanding the contribution of genetics to AIH, we carried out a case–control study to investigate the association between variants of *CARD10* rs6000782 and type 1 AIH in a Japanese population.

## Methods

### Study population

Consecutive type 1 AIH patients (n = 326) diagnosed according to the international diagnostic criteria for AIH [4] from the register of the Japanese National Hospital Organization (NHO) Liver Network Registry beginning in 2009 were enrolled in the present study as a multicenter cohort population [5]. Patients exhibiting primary biliary cirrhosis were excluded from the analysis. As controls, 214 healthy Japanese subjects (74 men and 140 women, with a mean age of 47.5 ± 10.8 years) with no history of liver disease were also enrolled. All patients did not have any other types of liver diseases such as chronic hepatitis C, alcoholic liver diseases, autoimmune liver diseases, or metabolic liver diseases. This study was conducted by adhering to the STOROBE statement (case–control studies). The study protocol was approved by the Ethics Committees of National Nagasaki Medical Center, and written informed consent was obtained from each individual.

#### DNA extraction and genotyping

**Fig. 1:**
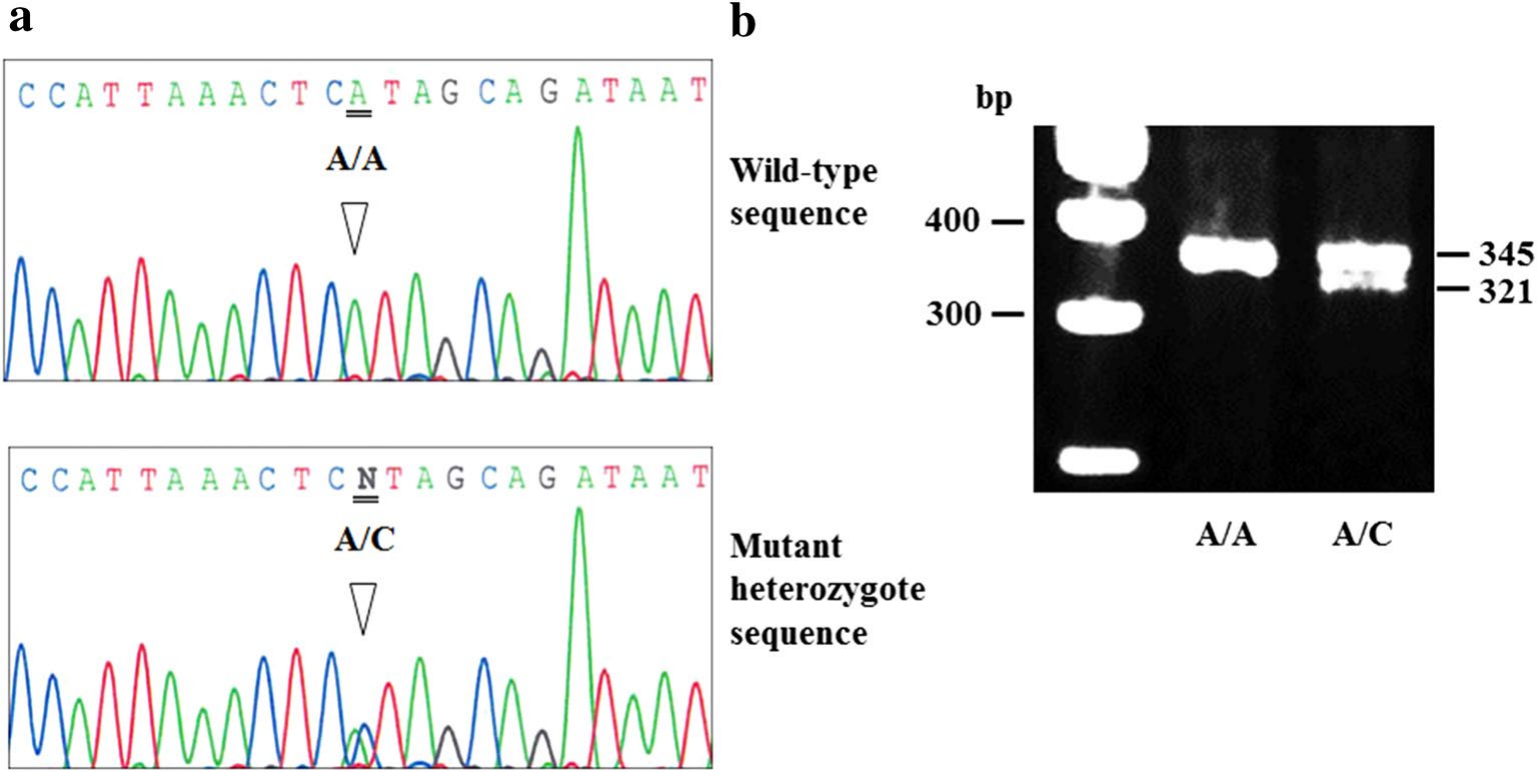
Genotyping of *CARD10* rs6000782. **a** *CARD10* rs6000782 is positioned 12,643 base pairs downstream in the 22q13.1 region. For sequencing primers, forward primer 5′-TTGAGACGGGGTCTCGCT-3′ and reverse primer 5′-GCCAAACCCGAGGTAATCTA-3′. **b** The SNP *CARD10* rs6000782 A/C were genotyped from a PCR fragment with an average size of 365 bp. The dimorphism rs6000782 A/C was typed by RFLP method, the amplified PCR product was digested with *X*spI restriction enzyme, resulting in 321 and 24 and 20 bp fragments for allele C and an intact fragment of 345 and 20 bp for allele A. The small fragments (<50 bp) are not visible on the gel

Blood samples were taken from all study participants, and genomic DNA was isolated from peripheral blood leukocytes using a DNA blood mini kit from Qiagen (Hilden, Germany) according to the manufacturer’s guidelines. *CARD10* rs6000782 genotypes were determined by the polymerase chain reaction–restriction fragment length polymorphism (PCR–RFLP) method. For the sequence (Fig. 1a), PCR products were treated with ExoSAP-IT (Affymetrix, Inc., Santa Clara, CA), and then sequenced using a BigDye Terminator v1.1 Cycle Sequencing Kit (Life Technologies, Tokyo, Japan). Sequences were analyzed using an Applied Biosystems 3130xl Genetic Analyzer (Life Technologies). Restriction fragment length polymorphism analysis was performed after the identification of single nucleotide polymorphism-specific restriction sites by NCBI Entrez SNP (http://www.ncbi.nlm.nih.gov/snp) and Takara Cut-Site Navigator (http://www.takara-bio.co.jp/enzyme). PCR restriction fragment length polymorphism genotyping to detect the 37928186A > C base pair change was performed using the following cycling profile: 95 °C for 5 min, followed by 35 cycles at 95 °C for 1 min, 56 °C for 1 min, and 72 °C for 1 min. The 365-base pair product was digested with *Xsp*I at 37 °C for 6 h and analyzed by 4 % NuSieve 3:1 agarose gel electrophoresis (Fig. 1b).

#### Statistical analyses

Results are expressed as mean ± SD. The statistical significance of differences between groups was calculated by either the Chi square test or Fisher’s exact test for categorical data and Mann–Whitney’s U test for quantitative data. Multivariate logistic regression analysis was performed with SPSS v.18 for windows (SPSS Statistics, Illinois). Deviation from Hardy–Weinberg equilibrium was assessed using the SNPAlyze software ver. 7.0 (Dynacom, Yokohama, Japan). A *p* value of <0.05 was considered significant.

## Results

### Demographic data of patients with AIH

Table 1 presents the demographic data of the subjected AIH patients. Among the enrolled type-1 AIH patients, 288 (88.6 %) were positive for ANA (>1:40) and 121 (38.2 %) for ASMA (>1:40). Among 326 eligible patients, 35 (10.7 %) had liver cirrhosis at the time of diagnosis, and among the remaining 291 patients without liver cirrhosis, 16 developed liver cirrhosis during the follow-up.

**Table 1:** Baseline characteristics of 326 Japanese AIH type 1 patients

Characteristics	N = 326 (%)
Female, n/total (%)	289/326 (88.7)
Age, years, mean ± SD	59.5 ± 13.3
Biochemistry	
AST, IU/L, median (IQR)	255.0 (92.5–723.0)
ALT, IU/L, median (IQR)	297.0 (104.5–818.0)
ALP, IU/L, median (IQR)	432.0 (316.5–584.0)
Total Bilirubin, mg/dl, median (IQR)	1.3 (0.8–4.6)
Albumin, g/dl, median (IQR)	3.9 (3.5–4.2)
IgG, mg/dl, median (IQR)	2239.0 (1809.5–2390.0)
Platelets, 10^4^/μl, median (IQR)	18.5 (13.9–23.0)
Serology	
ANA ≧ 1:40, n/total (%)	288/325 (88.6)
ASMA ≧ 1:40, n/total (%)	121/317 (38.2)
Histology	
Cirrhosis, n/total (%)	51/326 (15.6)
IAIHG criteria	
Score, median (IQR)	16 (14–18)
Probable AIH, n/total (%)	126/309 (40.8)
Definite AIH, n/total (%)	183/309 (59.2)

*AST* aspartate aminotransferase, *ALT* alanine aminotransferase, *ALP* alkaline phosphate, *IgG* immunoglobulin G, *ANA* anti-nuclear antibody, *ASMA* anti-smooth muscle antibody, *IQR* interquartile range, *IAIHG* International Autoimmune Hepatitis Group

### Association of *CARD10* polymorphisms with type-1 AIH

Genotype distributions were in Hardy–Weinberg equilibrium in cases and controls (Table 2). Genotype frequencies and distributions, as well as odds ratios (ORs) and 95 % confidence intervals (CIs) for the association with AIH are shown in Table 3. The rs6000782 C allele was shown not to be associated with an increased risk for AIH (OR 1.271; 95 % CI 0.747–2.161; *p* = 0.376).

**Table 2:** Frequencies of CARD10 rs6000782 genotypes

	Locus	Genotype	Observed number (%)	Expected number^a^	*p* value^b^
Patients with AIH					
n = 326	rs6000782	A/A	284 (87.1)	285.3	χ^2^ = 1.746
		A/C	42 (12.9)	39.3	*p* = 0.523
		C/C	0	1.4	
Healthy control					
n = 214	rs6000782	A/A	193 (90.2)	192.5	χ^2^ = 0.280
		A/C	20 (9.3)	20.9	*p* = 1.000
		C/C	1 (0.5)	0.6	

^a^Expected genotype frequencies based on observed allele frequencies and assuming Hardy–Weinberg equilibrium

^b^ *p* values were calculated using the Chi square test for Hardy–Weinberg equilibrium at individual loci

**Table 3:** CARD10 rs6000782 polymorphism in patients with type 1 AIH and healthy controls

	Healthy control (%) n = 214	AIH (%) n = 326	*p* value^a^	OR (95 % CI)
Genotype frequencies			0.186	
A/A	193 (90.2)	284 (87.1)		
A/C	20 (9.3)	42 (12.9)		
C/C	1 (0.5)	0		
Allele frequencies			0.376	
A	406 (94.9)	610 (93.6)		1
C	22 (5.1)	42 (6.4)		1.271 (0.747–2.161)

*OR* odds ratio, *CI* confidence interval

^a^Genotype frequencies were determined by χ^2^ test using 2 × 3 contingency tables between patients with AIH and healthy controls. Allele frequencies were determined by χ^2^ test using 2 × 2 contingency tables between patients with AIH and healthy controls

We also performed a detailed genotype–phenotype analysis using the clinical data. A detailed genotype–phenotype analysis using the clinical data revealed no significant association between rs6000782 and clinical findings of AIH patients (Table 4).

**Table 4:** Comparison of demographics between AIH patients with or without rs6000782 C allele

	rs6000782 C allele (+)	rs6000782 C allele (−)	*p*
	n = 42	n = 284	
Female, n (%)	38 (90.5)	251 (88.4)	0.465
Age, years, mean ± SD	59.6 ± 13.9	59.5 ± 13.3	0.786
Biochemistry			
AST, IU/L [median (IQR)]	189.0 (85.5–835.0)	264.0 (94.0–707.5)	0.620
ALT, IU/L [median (IQR)]	258.5 (93.0–906.0)	305.0 (104.5–813.0)	0.926
ALP, IU/L [median (IQR)]	402.5 (261.0–566.0)	437.0 (323.5–586.5)	0.242
Total bilirubin, mg/dl [median (IQR)]	1.9 (0.8–4.6)	1.2 (0.8–4.6)	0.680
Albumin, g/dl [median (IQR)]	3.9 (3.4–4.2)	3.9 (3.5–4.2)	0.844
IgG, mg/dl [median (IQR)]	2078.0 (1695.0–2691.5)	2250.0 (1835.5–2945.0)	0.199
Platelets, 10^4^/μl [median (IQR)]	19.6 (15.1–22.4)	18.4 (13.9–23.1)	0.712
Serology			
ANA ≧ 1:40, n (%)	36 (85.7)	252 (89.0)	0.339
ASMA ≧ 1:40, n (%)	13 (31.0)	108 (39.3)	0.301
Histology			
Cirrhosis, n (%)	8 (19.0)	43 (15.1)	0.515

*AST* aspartate aminotransferase, *ALT* alanine aminotransferase, *ALP* alkaline phosphate, *IgG* immunoglobulin G, *ANA* anti-nuclear antibody, *ASMA* anti-smooth muscle antibody, *IQR* interquartile range

## Discussion

AIH is characterized by an imbalanced regulation of the immune system in which innate and adaptive immune responses to hepatocyte antigens are important [6]. Genetic variation in the immune mechanisms that establish and maintain self-tolerance is likely to play a role in the development of AIH [7]. The susceptibility genes of AIH act alone or with environmental factors whose identity is mostly unknown [8]. The strongest association is with genes located within the HLA region, particularity those encoding the HLA-DRB1 alleles [2]. Up until now, evaluation of the non-HLA genetics of AIH has focused on small scale (usually non-replicated) candidate gene studies [9]. Genome-wide screening is a promising approach for the identification of the genetic determinants of complex diseases [8]. Large case–control studies with genome-wide surveys of genetic risk have been demonstrated for primary biliary cirrhosis (PBC) [10, 11]. AIH was subjected to the a similar genome-wide survey. The first application for type 1 AIH by de Boer et al. in AIH patients identified three genes exhibiting significant association in 649 patients and 13,436 healthy controls in Dutch and German populations. The main finding of this study was the strength of the AIH association with HLA, although it also identified associations with the *SH2B3* rs3184504 *A allele and *CARD10* rs6000782 *C allele.

The present study found no association of *CARD10* rs6000782 variants with type 1 AIH in a Japanese population. The major strength of this study is the finding that the association discovers by deBoer in a Caucasian Northern European Dutch and German population is not generalizable to the East Asian Japanese population. While this contrasts with the finding of de Boer et al. in a Caucasian population, it is in agreement with the documented lack of association between *CARD10* variants and AIH in populations of diverse racial backgrounds. Gene–gene interactions or epistasis have been proposed to occur between genes that cluster within specific immune pathways, thus enhancing their effect on disease susceptibility [12]. These have been reported in autoimmune diseases, including a possible interaction between HLA and non-HLA genes [13]. This suggests that further gene–gene interaction studies will be necessary to determine the associations of different susceptibility loci in AIH.

The main finding of the study by de Bore et al. [3] remains the relative strength of the HLA associations. The associations of AIH with variants of *SH2B3* rs3184504 (*p* = 7.7 × 10^−8^) and *CARD10* rs6000782 (*p* = 3.0 × 10^−6^) did not reach the accepted level of significance required to declare genome-wide significance (*p* < 5 × 10^−8^). However, the prior association of *SH2B3* variants with autoimmune diseases suggests that this association is likely to be confirmed with larger cohorts. We did not analyze the association of *SH2B3* rs3184504 and AIH in our current study because it is almost monomorphic in a Japanese population (http://www.ncbi.nlm.nih.gov/projects/SNP/snp_ref.cgi?rs=3184504). However, such validation is necessary because attempts to generalize genetic associations across ethnicities have had mixed results. For *CARD10* rs6000782, more validation studies are warranted. Our study only enrolled patients with a definite diagnosis of AIH, in order to prevent any potential case ascertainment bias. This approach limited the sample size and therefore introduced the limitation of reduced statistical power, which might have prevented us from identifying potential associations between *CARD10* rs6000782 and AIH.

## Conclusions

Our findings showed that *CARD10* rs6000782 is not likely to be associated with type 1 AIH, at least in a Japanese population. However, because allele frequency distributions differ according to ethnicity, replication in other populations and functional studies should be initiated in order to clarify the contribution of this genetic background in the development of AIH . Genetic variations associated with AIH susceptibility remain for further investigation.

## Abbreviations

AIH: autoimmune hepatitis
CARD10: caspase recruitment domain-containing protein 10
HLA: human leukocyte antigen
NHO: National Hospital Organization
SH2B3: SH2B adaptor protein 3
SNPs: single nucleotide polymorphisms
